# Dual-stage Acting Dendrimeric Nanoparticle for Deepened Chemotherapeutic Drug Delivery to Tumor Cells

**DOI:** 10.34172/apb.2024.054

**Published:** 2024-06-29

**Authors:** Mohammad Shahpouri, Mohammad Amin Adili-Aghdam, Hossein Mahmudi, Saeedeh Ghiasvand, Hamed Dadashi, Aysan Salemi, Sajjad Alimohammadvand, Leila Roshangar, Abolfazl Barzegari, Mehdi Jaymand, Rana Jahanban-Esfahlan

**Affiliations:** ^1^Department of Medical Biotechnology, Faculty of Advanced Medical Sciences, Tabriz University of Medical Sciences, Tabriz, Iran.; ^2^Department of Biology, Faculty of Science, Malayer University, Malayer, Iran.; ^3^Stem Cell Research Center, Tabriz University of Medical Sciences, Tabriz, Iran.; ^4^Nano Drug Delivery Research Center, Health Technology Institute, Kermanshah University of Medical Sciences, Kermanshah, Iran.; ^5^Student Research Committee, Kermanshah University of Medical Sciences, Kermanshah, Iran.

**Keywords:** Hypoxia, Dual-stage acting, Dendrimeric NPs, Deep penetration, Drug delivery, Residual tumor cells

## Abstract

**Purpose:**

We report on the design of hypoxia-induced dual-stage acting dendrimeric nanoparticles (NPs) for selective delivery of two chemotherapeutic model drugs doxorubicin (DOX) and tirapazamin (TPZ) for deepened drug delivery into hypoxic tumors *in vitro*.

**Methods:**

PAMAM G5 dendrimers were crosslinked with a hypoxic azo linker, attached to a mPEG to form a detachable corona on the dendrimer surface (PAP NPs). NPs were characterized by Zeta sizer, transmission electron microscope (TEM), Fourier transforms infrared (FTIR) and drug release kinetics. The anti-cancer performance of PAPs was evaluated by numerous tests in 2D and 3D cultured MDA-MB-231 breast cancer cells.

**Results:**

MTT assay showed a significant difference between PAP and PAMAMG5 in terms of biocompatibility, and the effect of PAP@DOX was significantly greater than free DOX in hypoxic conditions. The results of DAPI and Annexin V-FITC/PI cell staining also confirmed uniform drug penetration as validated by induction of 90% cell apoptosis in spheroids and a high level of PAP@DOX-induced ROS generation under hypoxia conditions. Mechanistically, PAP@DOX significantly reduced the expression of mTOR, and Notch1, while the expression of Bax and Caspase3 was considerably unregulated, compared to the controls. Importantly, hypoxia-responsive disintegration and hypoxia-induced activation of HAP drug were synergized to promote deep and homogenous HAP distribution in whole microtumor regions to efficiently eliminate residual tumor cells.

**Conclusion:**

Our results indicate the safety and high therapeutic potential of PAP system for targeted drug delivery of chemotherapeutics in particular HAPs which show maximum anti-cancer activity against hypoxic solid tumors.

## Introduction

 Breast cancer is the second most common cancer in the world. The most predisposing factors to breast cancer are sexual hormones, age, familial history, and long-term radiation exposure. Depending on ER, PR and HER2/neu receptors, breast cancer is divided into four subgroups luminal A, luminal B, triple negative (TNBC) and triple positive. Unlike their initial success, available treatment methods including surgery, chemotherapy, radiation and endocrinal therapy have faced side effects and clinical challenges.^1,2^ So, focuses were placed on molecularly targeted therapies using nano drug delivery systems (NDDSs) such as liposomes,^3^ niosomes,^4,5^ polymeric NPs,^6-8^ hydrogels,^9,10^ metallic NPs,^11,12^ exosomes,^13,14^ and biomimetic nanoparticles (NPs),^15^ among others. Despite the diversity in the design of NDDSs, controlling drug release is deemed successful. To tackle this, smart and multi-sage acting NPs emerged which take advantage of specific tumor microenvironment features such as temperature, acidity, hypoxia, redox, presence of specific enzymes and so on for their self-activation only upon localization in the desired site.^16-18^

 Hypoxia as one of the key hallmarks of tumor microenvironment is a key barrier for efficient drug delivery. Hypoxic cells are deeply embedded within tumors in the middle and central layers far from the blood vessels and, thus are inaccessible to systematically administered therapeutics and NPs.^19,20^ Plus, hypoxia leads to drug resistance and increased proliferation of cancer cells via disturbing the key cellular signaling pathways involved in tumor stemness features, dormancy and metastatic spread. So, ideal NDDS should be able to drill into tumor core, specifically and homogenously deliver the drug into the hypoxic core in a controlled manner.^3,21-24^ In this regard, one can use a hypoxia-responsive linker in the design of NPs or a hypoxia-activating prodrug (HAP) such as Tirapazamin (TPZ) which shows optimal and enhanced function under hypoxia than normoxia condition.^25,26^ Meanwhile, in the design of hypoxia-responsive nanocarrier, small polycationic NPs such as chitosan, dendrimers and polyethylene imine (PEI) are good candidates benefiting from a high penetration efficiency into the biological membranes.^27^ In particular, poly(amido)-amine (PAMAM)-dendrimer can also perform endosomal escape through the “proton sponge effect” and in the same way can act as a multi-drug loading carrier. However, toxicity issues and capture by non-target cells such as immune cells should yet be avoided.^28^ To resolve this, one study reported iCluster NDDS based on dendrimers which are coated onto PCL-CDM, PEG- b-PCL, and PCL by nanoprecipitation method using a pH-cleavable amide linker and can shift their size from ~100 nm to ~5 nm selectively upon localization into hypoxic 4T1 breast tumors for cisplatin delivery and pose potent anti-metastatic potential with more than 110 days tumor-free survival in mice.^29^ In another formulation, a dendrimeric NP with a size of ~200 nm and hypoxia-responsive size-reducing potential is used for the delivery of HIF-1α-siRNA and doxorubicin (DOX) to hypoxic breast cancer cells.^30^ Likewise, self-assembled PEG and poly[glutamic acid (3-(2-nitro-imidazolyl)-propyl)] are used as a hypoxia-responsive NDDS for selective delivery of DOX.^31^ Other formulations have used hypoxia-responsive NPs for selective delivery of HAPs.^32^ Recently, our group formulated hypoxia-responsive chitosan NPs for fingolimod delivery, which achieved superior performance in inhibiting highly-proliferative 4T1 tumors in mice.^18^

 With this in mind, we formulated a hypoxia-responsive multi-stage acting dendrimeric system with good stability, high safety profile and desirable drug loading content for specific and controlled delivery of two model drugs DOX and TPZ into hypoxic triple-negative breast cancer (TNBC) tumor cells. A single low dose of PAP@TPZ2 was capable of homogenous distribution and widespread induction of apoptosis and destruction of large hypoxic MDA-MB-231 spheroids *in vitro*.

## Materials and Methods

###  Materials

 Bi-functional 4,4-dicarboxylic Azo linker (AZ) with 300Da MW was purchased from TCI, USA. Methoxy PEG (NHS-PEG-methoxy, MW 3000Da), 1-Ethyl-3-[3-(dimethylamino) propyl] carbodiimide (EDC), N-hydroxysuccinimide (NHS), Generation 5 polyamidoamine (PAMAM G5) dendrimer (28.8 kDa), cellulose dialysis bag (cut off 12-14 kDa), 4,6-diamidino-2-phenylindole dihydrochloride (DAPI), 2′,7′-Dichlorofluorescin diacetate (H2DCFDA), Annexin-V-FITC Pi KIT, phosphate buffered solution (PBS), 3-(4,5-dimethyl-2-thiazolyl)-2,5-diphenyl-2Htetrazolium bromide (MTT), and Trypsin-EDTA, and TPZ were obtained from Sigma-Aldrich St. Louis, MO, USA. The source of other used materials was as follows: Doxorubicin hydrochloride (Azista Industries Pvd Ltd), RPMI1640 and FBS (AnnaCell, ScienCell, USA), Pensterp solution (P/S, ScienCell, USA), Rhodamin 123 (Dojindo, Japan), Agarose powder, DNA safe stain and RNAX Plus solution (Cinnagene Co, Iran), SYBR^TM^ Green real Time PCR master mix (RealQ Plus 2x Master Mix Green, Ampliqon), First strand cDNA synthesis kit (Thermo Fisher, Waltham, Massachusetts, USA). Primers were synthesized by CinaClone, Iran. All other reagents and solvents used in this work were analytical grade and were obtained from Merck.

###  Nanoparticle preparation 

####  Nanoparticle synthesis 

 20mg of azo-linker was added to 1 mL pyridine on the stirrer. Then, 20 μL of EDC and 12μL of NHS were used to activate the carboxyl groups for 2 hours by stirring. Thereafter 100 mg of Methoxy polyethylene (mPEG) were solved in 5mL water and added to the solution using a syringe pump instrument on the stirrer for 24 hours. The solution was then introduced to an evaporator (50 °C) to remove solvent phases, and then heated for 24 hours in the oven (40 °C). After that, 6 mL dichloromethane solvent was added and vortexed. Then, 5 mL of diethyl ether was added and centrifuged to purify and precipitate free Azo. Supernatants were collected into a falcon tube under the laminar hood. The reaction between PEG-AZO-COOH and PAMAM-NH2 in PBS (pH = 7.4) was promoted by stirring at room temperature for 24 hours. To this, 40 mg of mPEG-azo was suspended in 2 mL of PBS (pH = 7.4). Finally, 70 μL of PAMAM G5 dendrimer (5 wt. % in methanol) was added to the mPEG-AZO and placed on the stirrer overnight. The final product was dialyzed against PBS using Amicon Ultra tubes (MWCO 10000) to obtain mPEG-AZO-PAMAM.

####  Drug loading 

 Two milligrams of DOX or 1 mg TPZ were dissolved in methanol and mixed physically with mPEG-dendrimer overnight in the dark. For the removal of unloaded drugs, dialysis was performed twice.

####  Encapsulation efficiency 

 Two milliliters of PAP@DOX or PAP@TPZ was poured into 12 kDa dialysis bag and placed into a flask containing PBS. After 30 minutes, the absorbance of the drugs was read using a spectrophotometer (Cecil BioAquarius CE 7250, England). Then, the amount of unloaded drug concentration is calculated according to drug standard curves. Eventually, encapsulation efficacy and drug loading percentage are obtained from the following formula:


$$Drug\;encapsulation\;efficiency\left(\%\right)=\frac{initial\;drug\;concentraion-unload\;drug\;concentraion}{initial\;drug\;concentraion}*100$$

####  Drug release studies

 The release profile of drugs from PAP NPs was performed by dialysis bag diffusion method. To this, 2 mL of each NP was dialyzed in 100mL PBS solution with pH 7.4 and pH 5.4 on a shaker incubator at 37 °C. Then 2 mL of samples were withdrawn at specific intervals and to maintain sink condition same amount of PBS was added. The absorbance of samples was read separately for DOX (λmax = 485 nm) and TPZ (λmax = 450 nm). Finally, concentrations of released drugs were obtained from standard curves. We also studied drug release kinetics of both drugs by mathematical models including Zero-Order, First-Order, Higuchi, Hixson-Crowell and Peppas Korsmeyer.

###  Nanoparticle characterization 

####  Fourier transforms infrared (FTIR) spectroscopy

 FTIR analysis was conducted in reference to our previous works.^33-36^ Briefly, to investigate the chemical structure of PAP by FTIR, 1 mg of mPEG-Azo, and PAMAM G5-mPEG-Azo samples were mixed with potassium bromide (KBr), and the mixture was compressed in a disk and evaluated in the IR range of 400-4000 cm^-1^ using Fourier transform infrared spectrometer (FTIR 4300, Schmatz, Japan).

####  Particle size distribution and zeta potential

 Particle size, poly dispersity index (PDI) and surface charge of NPs were measured by dynamic light scattering (DLS) and zeta sizer at 25 °C (Zetasizer Nano ZS90, Malvern Instruments, Malvern, UK).

####  Size and morphology of PAMAM- mPEG-Azo

 The morphology, topography, size and distribution of PAP NPs were further studied using transmission electron microscope (TEM).

###  2D cell culture 

 MDA-MB-231 cell line (Pasteur Institute of Iran, Tehran) were cultured in RPMI supplemented with 10% FBS, streptomycin and penicillin in an incubator at 37 °C with 90% humidity and 5% CO_2_. When cells reached 75% confluency, cells were trypsinized and counted using trypan blue staining for further studies.

###  MTT assay 

 The cytotoxicity and biocompatibility of PAP and PAP@Dox on MDA-MB-231 cell line were evaluated by MTT assay under normoxia and hypoxia conditions. For this purpose, firstly, each well of the plate was filled with 200 µL of the complete culture medium containing 6000 cells. After 24 hours incubation, the cells were treated with 0, 10, 20, 30, 40 concentrations of free DOX, PAMAM@DOX, PAP@DOX, PAMAM, PAP. Then the treated cells were incubated for 24 hours in a 37 °C incubator. Then, 50 µL of 2 mg/mL MTT solution was added to each well. Untreated cells were considered as negative controls. After 4 hours, the media was carefully removed and 200 μL of DMSO was replaced. Then, the plates were incubated for 60 minutes at 37 °C and the absorbance of formazan was measured at 570 nm using an ELISA reader device (Bioteck). The relative toxicity was obtained by comparing the percentage of living cells to controls in quadruplets.

 To evaluate the efficiency of PAP@DOX under hypoxic conditions, we used a hypoxic incubator with < 1% oxygen. After the cells reached 80% confluence, the cells were counted by trypan blue staining and 10 000 cells were added to each well of the 96-well plate. After 24 hours, the cells were incubated for 6 hours in a hypoxia incubator and then drug treatment was done for DOX and PAP@DOX (0, 2.5, 5, 10, 20 μg/mL).

###  3D cell culture and tumor spheroid formation

 Multicellular tumor spheroid was formed by culture of 20 000 MDA-MB-231 cells on 1% agarose embedded 96-well plates for 24 hours. Then, spheroid size and morphology are monitored by an optical microscope every day. To evaluate PAP@DOX, PAP@TPZ and free DOX penetration into hypoxic spheroid core, spheroids were treated with PAP@DOX for 24, 48 and 72 hours. Eventually, size and morphology changes were assessed by an optical microscope (Olympus, Japan).

###  DAPI staining 

 DAPI staining was used to further evaluate apoptotic cell changes in MDA-MB-231 spheroids treated with PAP and PAP@DOX for 24 hours. Then, cells in 96-well plates were washed with PBS and fixed with 4% paraformaldehyde for 10 minutes and then washed with PBS three times. Next, cells were treated with 0.1% Triton X100 for 10 minutes, and again washed with PBS three times. Finally, DAPI dye was added and plate was incubated in the dark for 3 minutes. The results were visualized with Citation V cell imaging system (Bioteck).

###  Annexin-FITC/PI staining 

 Annexin-FITC staining (green color) and propidine iodine (red color) was used to determine the amount of live (pre-apoptotic) cells and dead (apoptotic) cells, in 2D and 3D cultured tumor cells respectively. For this, cells with a density of 2 × 10^5^ cells were cultured in a 24-well plate and treated with PAP@DOX and DOX for 24 hours. After that, cells were washed twice with PBS and then stained with 5 µL of Annexin V-FITC and 2 μL of PI dye for 10 minutes. Results were analyzed using Citation V cell imaging system with filters of 515-545 nm for FITC and 535-617 nm for PI.

###  DCFH test

 ROS generation was measured using H2DCFDA as ROS marker. For this purpose, MDA-MB-231 cells were cultured in 21% normoxia and 1% hypoxia in 24-well plates and after reaching the appropriate density, 5 × 10^3^ cells/well were seeded and 24 hours later were treated with PAP@DOX for 24 hours. Then, treated with 10 µM H2DCFDA in RPMI-1640 medium for 24 hours at 37 °C. Next, the supernatant was discarded and the cells were washed 3 times with PBS. Finally, the reduction of DCF substance and the fluorescence intensity were immediately read at wavelengths of 475-570 nm by a microplate reader (BioTek, Winooski, VT, USA). Data were expressed as mean fluorescent intensity (MFI) calculated by ImageJ software.

###  Reverse transcription (RT) real-time PCR 

 To this end, total cellular RNA from MDA-MB 231 spheroids treated with PAP@DOX2 as well as untreated cells as controls was isolated using RNAX Plus solution (Cinagene, Iran) according to the manufacturer’s instructions. The quantity and quality of total RNA was evaluated using Nanodrop (Thermo Scientific, USA). Reverse transcription of isolated RNAs (1 μg) was performed by a cDNA Synthesis Kit. And, finally real-time PCR reactions were performed using Master SYBR Green according to our previous works.^37^ PCR reaction was set on a real-time PCR instrument (96 Light Cycler, Roche, Germany) at 95 °C for 3s, followed by 45 cycles at 95 °C for 5 seconds and 60 °C for 30s. Specific relative mRNA expression levels of genes were normalized to the endogenous control gene GAPDH. The sequence of primers is shown in Table 1. Finally, the ΔΔCt method was used for data analysis using the following formula:

 Fold change = 2^-ΔΔCT^

**Table 1:** Sequence of primers for RT real-time PCR

**Primer**	**Primer sequence (5′-3′)**	**Product size**
Notch1	F:ACAAGGTGTCTTCCAGATCCT R:CTTGCCCAGGTCATCTACGG	169
GAPDH	F:CAAGCTCATTTCCTGGTATGACA R:GGGAGATTCAGTGTGGTGGG	189
mTOR	F:ACTGGAGGCTGATGGACACAAA R:CCGTTTTCTTATGGGCTGGC	132
Bax	F:ATCCAGGATCGAGCAGGGCG R:GGTTCTGATCAGTTCCGGCA	162
Casp3	F:TGCCTGTAACTTGAGAGTAGATGG R:CTTCACTTTCTTACTTGGCGA TGG	115

###  Statistical analysis 

 Data were presented as mean ± standard deviation (SD) and were analyzed by GraphPad Prism version 9.0. Student’s t-test and two-way analysis of variance (ANOVA) followed by post hoc Tukey test was used to analyze the difference among experimental groups. All experiments were performed at least as duplicates. Values of *P* < 0.05 were considered statistically significant.

## Results and Discussion

###  Characterization of hypoxia-sensitive nanoparticles 

**Figure 1:**
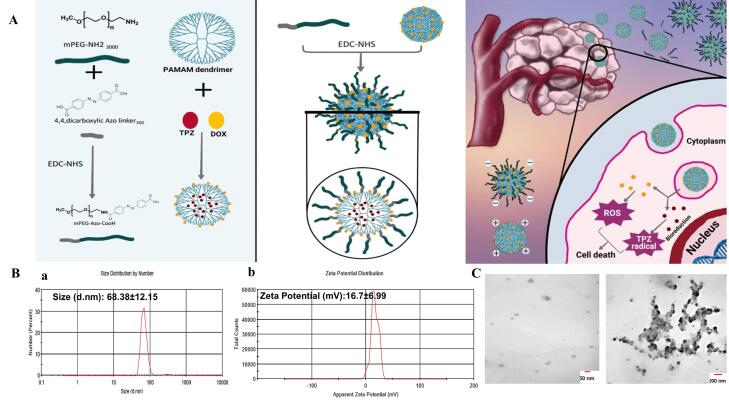
Nanoparticle synthesis and characterization. **A,** Schematics of PAP preparation and working principle. **B,a**. DLS and **b,** zeta potential and **C,** TEM images of PAP NPs

**Figure 2:**
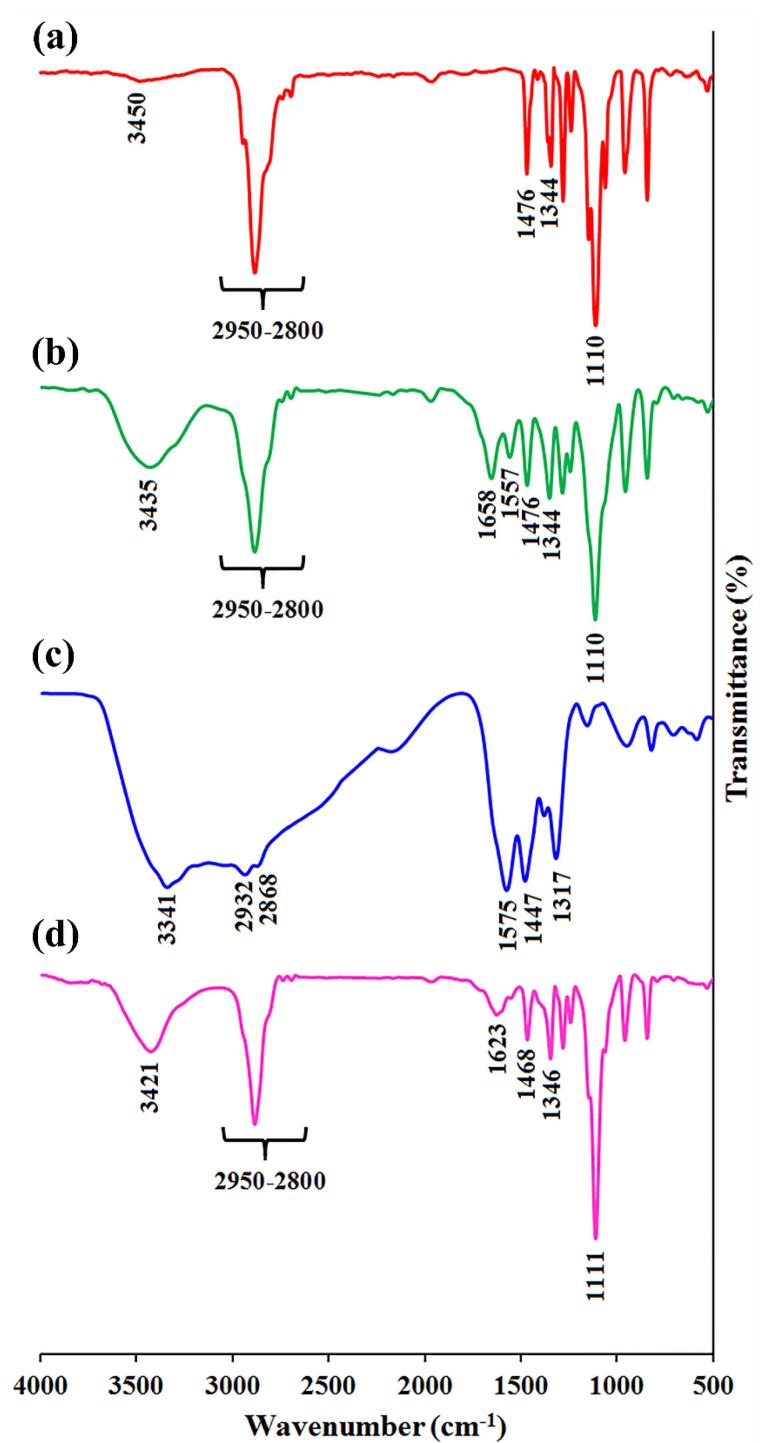
The FTIR spectra of **a,** mPEG, **b,** mPEG-Azo, **c,** PAMAM, and **d,** PAMAM-g-mPEG

 PAMAM dendrimer G5 is reported to exhibit a size of ~5.4nm and a high positive charge of ~ + 50.03 mV among G0-G7 generations.^38^ DLS results indicated that PAP NPs were successfully synthesized with a size of 68.38 nm and zeta potential was reduced to + 16.7mV (Figure 1A). In line with this, TEM images confirmed a size of ~50 nm with spherical morphology for PAP NPs (Figure 1B). Drug encapsulation efficiency of TPZ and DOX was determined 75.4% and 78.6%, respectively. The successful functionalization of mPEG with azobenzene-4,4’-dicarboxilic acid linker as well as grafting of mPEG chains onto PAMAM was verified using FTIR spectroscopy as shown in Figure 2. The FTIR spectrum of the mPEG was characterized by the stretching vibration of terminal hydroxyl group at 3450 cm^-1^, the stretching vibrations of aliphatic C—H groups at 2950 to 2800 cm^-1^, the bending vibrations of C—H groups at 1344 and 1467 cm^-1^, and the sharp and strong band at 1110 cm^-1^ related to the stretching vibration of C—O group (Figure 2a). The appearance of new adsorption bands at 1557 and 1658 cm^-1^ related to the stretching vibrations of aromatic C = C and carboxyl groups, respectively, that confirmed the successful functionalization of mPEG with azobenzene-4,4’-dicarboxilic acid moiety (Figure 2b).

 The FTIR spectrum of the PAMAM exhibited the stretching vibrations of various amides (I, II, and III) at 1317, 1447, and 1575 cm^-1^, the stretching vibrations of aliphatic C—H groups at 2932 and 2868 cm^-1^, and the broad band centered at 3341 cm^-1^ related to the amine terminal groups (Figure 2c). After grafting of mPEG onto PAMAM, the FTIR spectrum showed combination adsorption bands of PAMAM and mPEG as seen in Figure 2d. The most important adsorption bands in the FTIR spectrum of the PAMMAM-g-mPEG are the stretching vibrations of remained amine terminal groups and adsorbed water molecules at 3421 cm^-1^, the stretching vibrations of various aliphatic C—H groups at 2950 to 2800 cm^-1^, the conjugated esteric carbonyl group at 1623 cm^-1^, the stretching vibrations of various amides at 1468 and 1346 cm^-1^, and the sharp and strong band at 1111 cm^-1^ corresponded to the stretching vibration of C—O groups.

 Considering numerous biological barriers in the way of perfect drug delivery to tumor cells,^16^ hypoxia-responsive NPs can be designed with a size of less than 10 nm for better permeability, however, the risk of quick elimination through renal filtration should also be avoided. In our study, we introduce a multi-stage acting NP in which the core of NPs is ultrasmall (5 nm) highly-charged PAMAM dendrimer which after 1:1 pegylation, its size increased to 68 nm meanwhile the zeta potential was modulated to + 16 mV. These features made PAP stable and compatible, however upon localization into the hypoxic core of large TNBC microtumors, PEG shell is decomposed due to bioreduction of azo linker, producing ultrasmall particles which rapidly cross and penetrate the cellular membrane, escaping endosome and efficiently delivery chemotherapeutic drug DOX and TPZ to the nucleus for efficient ROS generation and induction of cell apoptosis. In a similar study, Xie et al reported on the design of PAP NPs for combined DOX and anti-HIF siRNA delivery. Authors find 20% pegylation better than 10% pegylation in terms of better cell distribution and stability. After loading with DOX, the size of the final NPs was 254 ± 72 nm, with a surface charge of 35 ± 3mV.^30^ In our study, the size and zeta potential of our NPs was only moderately modified due to less ratio of pegylation (1:1), yet we obtained improved stability, decreased toxicity of PAP upon cancer cells and superior performance of PAP@DOX by single agent anti-cancer therapy in large TNBC spheroids compared to 180μm reported by Xiang et al.^30^

###  Drug release profile

**Figure 3:**
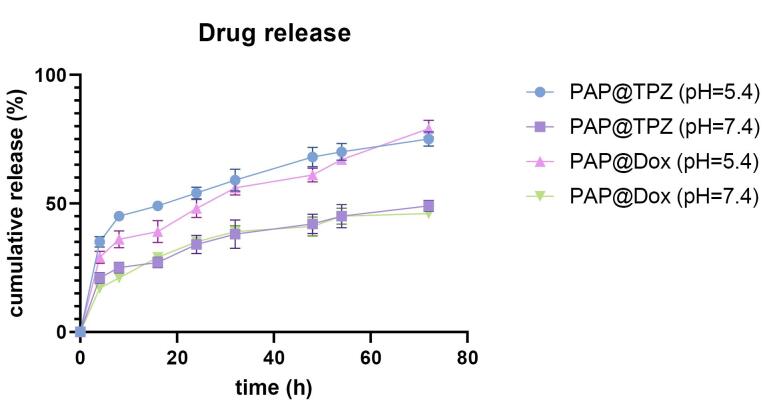
Drug release behavior of PAP@DOX and PAP@TPZ NPs

 The drug release profile of PAP@DOX and PAP@TPZ under pH = 7.4 and pH = 5.4 showed a sustained drug release behavior for both NPs, where under acidic conditions the drug release profile was markedly higher compared to the normal condition (physiologic pH) for both NPs. Under acidic conditions, a sustained drug release of ~79% and ~75% was recorded for PAP@DOX and PAP@TPZ, respectively by 72 hours (Figure 3).

 For drug release kinetics, the best-fitted model with the highest regression coefficient (R^2^) close to 1 was considered. Accordingly, for PAP@TPZ and PAP@DOX under physiological and acidic pH the best models were Higuchi and Korsmeyer-Peppas, while Higuchi describes the drug release by diffusion, Korsmeyer–Peppa model describes drug release from a polymeric NP.^5,39^ The calculated regression coefficient values are shown in Table 2.

**Table 2:** Drug release kinetics of PAP@DOX and PAP@TPZ.

**Kinetic model**	**Equation**	**DOX**		**TPZ**	
		**pH=7.4**	**pH=5.4**	**pH=7.4**	**pH=5.4**
Zero-order	$F=k_{0}t$	R^2^ = 0.756	R^2^ = 0.768	R^2^ = 0.752	R^2^ = 0.690
First order	$Ln\left(1-F\right)=-k_{f}t$	R^2^ = 0.845	R^2^ = 0.880	R^2^ = 0.823	R^2^ = 0.843
Higuchi	$F=k_{H}\sqrt{t}$	R^2^ = 0.968	R^2^ = 0.949	R^2^ = 0.940	R^2^ = 0.908
Hixson-Crowell	$1-\sqrt[{3}]{1-F}=k_{1/3}t$	R^2^ = 0.828	R^2^ = 0.846	R^2^ = 0.800	R^2^ = 0.795
Korsmeyer-Peppas	$F=k_{kp}t^{n}$	R^2^ = 0.991	R^2^ = 0.969	R^2^ = 0.964	R^2^ = 0.955

###  Cytotoxicity assay

 Breast cancer is one of the most common cancers, and is the second primary cause of death in the world. TNBC is a type that lacks or has low levels of estrogen receptor (ER), progesterone receptor (PR) and human epidermal growth factor receptor 2 (HER2). TNBC accounts for 15-20% and is the most common type of breast cancer to be treated.^40^ Common treatments are surgery, radiation therapy, and chemotherapy, but today, more attention is paid to targeted treatments that are associated with fewer complications. One of these methods is the application of smart nanocarriers that can operate in the conditions of the tumor microenvironment, and not the other healthy organs or normal cells in the body or blood circulation.^41,42^ In our study, we employed MDA-MB-231 cancer cells cultured as 2D monolayer as well as 3D mammospheres to evaluate anti-cancer potential of hypoxia-responsive dendrimeric nanosystems.

**Figure 4:**
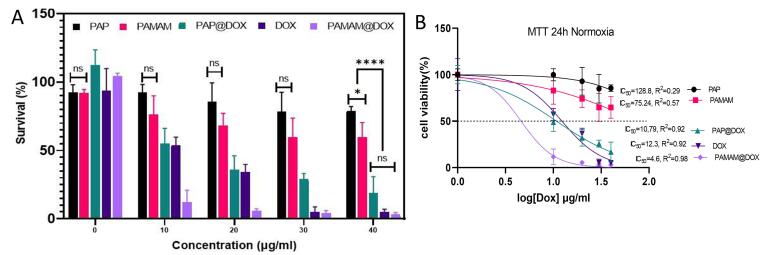
2D cell culture under normoxia conditions. **A,** cell survival and **B,** IC_50_ values of PAP NPs

 MTT assay was performed under normoxia and hypoxia 2D cell culture conditions. Under normoxia incubator, PAMAM dendrimer showed toxicity at 10-40 μg/mL doses. The cytotoxicity profile for studied groups was in the order of PAMAM@DOX > free DOX > PAP@DOX > PAMAM > PAP (Figure 4a). The IC_50_ value was calculated as 128.8μg (PAP), 75.24 μg (PAMAM), 10.79 μg (PAP@DOX), 12.3 μg (DOX) and 4.6 μg (PAMAM@DOX) (Figure 4b). Accordingly, for all doses ranging from 10-40 μg/mL, more than 80% cell viability was achieved for PAP, indicating the good biocompatibility of developed PAP NPs compared to PAMAM. PAMAM@DOX surpassed DOX and PAP@DOX at doses 10, 20, 30 μg/mL. However, at the highest dose of 40 there was no statistically significant difference among PAP@DOX, DOX and PAMAM@DOX (*P* < 0.05) (Figure 4a).

**Figure 5:**
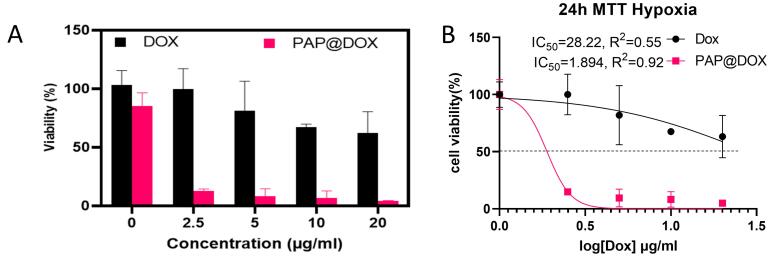
2D cell culture under hypoxia conditions. **A,** cell survival and **B,** IC_50_ values of PAP NPs

 Under hypoxic environment, lower doses of PAP@DOX NPs were capable of reducing cell viability down to ~20% and 10% at all doses. Meanwhile, cell sensitivity to DOX was significantly reduced under hypoxia conditions compared to normoxia. These results show the hypoxia-responsive potential of PAP for self-activation under hypoxia conditions (Figure 5a). Also, IC_50_ value was archived at lower doses of ~1.8 μg for PAP@DOX, ~6 times lower than the dose calculated for DOX (Figure 5b).

 When it comes to drug delivery into the dense and hypoxic microenvironment of solid tumors, NPs with a small size and positive charge are favorable ones.^43^ In this line cationic polymers such as chitosan, PEI and albeit branched spheric-shaped polymers such as dendrimers are top options. In our unpublished study, we find that PAMAM has remarkably high penetration depth and transfection ability of chemotherapeutics than low molecular weight chitosan, however, in terms of compatibility, chitosan is a better option. Different generations of PAMAM each display different transfection abilities. We used the G5 dendrimer with 128 free amine groups, and highest positive charge among G0-G7, and thus expected to have more toxicity upon cellular membrane at higher concentrations.^38,44^ In our study, we found that PAMAM G5 was non-toxic to cells at concentrations of 10 to 20 μg/mL, but at higher concentrations, toxicity was observed due to the strongly positive charge of the amine groups. This toxicity was reduced by pegylated dendrimer (PAP) which produced ~100% cell viability for doses (10-30μg) and more than 70% at the highest studied dose (40 μg/mL).

###  ROS activity

**Figure 6:**
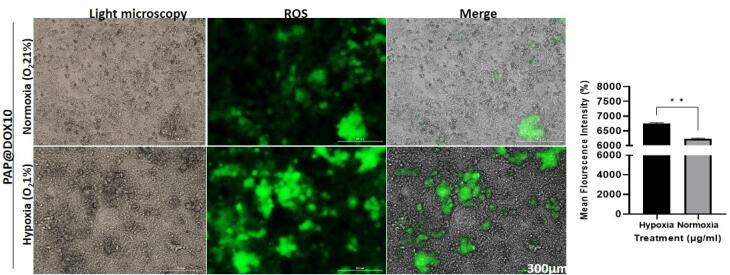
ROS activity of PAP@DOX under normoxia and hypoxia conditions

 DCFH test was performed to measure ROS generation potential of PAP@DOX under hypoxia and normoxia conditions. We observed that PAP@DOX was capable of more ROS generation, validated by a higher fluorescence intensity achieved in hypoxia (6758.435 ± 25) *vs.* normoxia (6230.607 ± 23) conditions (*P* < 0.05) (Figure 6).

###  Live/dead staining

**Figure 7:**
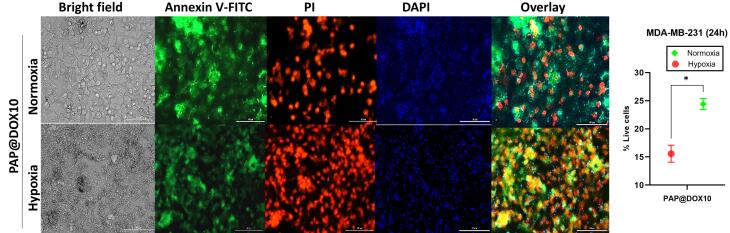
Live/dead staining of PAP@DOX-treated cells in 2D culture under normoxia and hypoxia conditions

**Figure 8:**
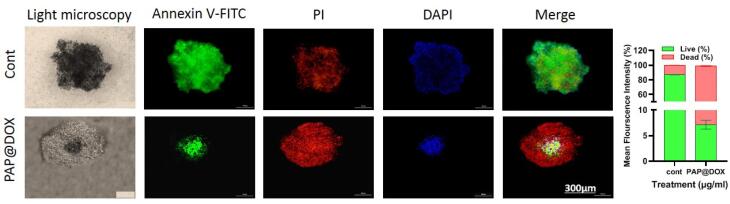
Live/dead staining of PAP@DOX-treated cells in 3D tumor spheroids

 Annexin FITC/PI staining which reveals proapototic and apoptotic cells as live and dead cells, respectively was performed on two-dimensional cell culture under hypoxia and normoxia conditions as well as tumor spheroids. In line with MTT assay results, we found that the anti-cancer efficacy of cancer cells treated with PAP@DOX10 NPs was significantly enhanced under hypoxic conditions than normoxia, reducing the number of live cells to 24.5% and 15.57% under hypoxia and normoxia conditions, respectively (*P* < 0.05) (Figure 7). As a better mimetic of tumor hypoxia, live/dead staining was also performed on MDA-MB-231 tumor spheroids. The results showed that after 72 hours the number of live cells in the untreated group was more than 86.94%, while PAP@DOX treatment decreased cell viability to 7.77% (Figure 8). In contrast to 2D cell culture, there was a sharp and obvious difference between control and PAP@DOX-treated hypoxic tumor spheroids, showing their potential as reliable models of tumor hypoxia.

###  DAPI staining

**Figure 9:**
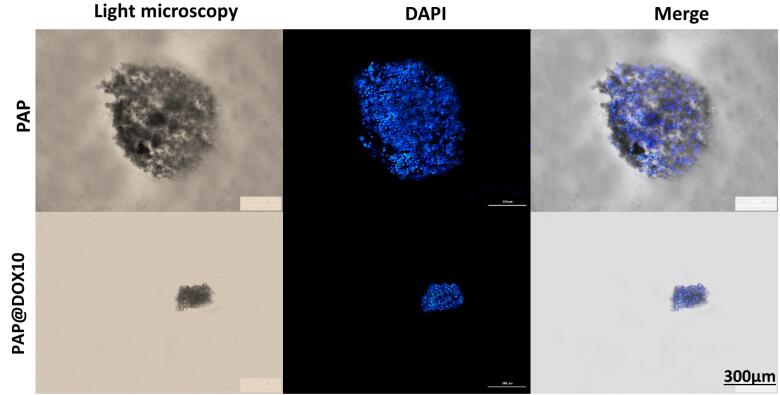
The anti-cancer effect of PAP@DOX on tumor spheroids

 We further used DAPI staining for better visualization of PAP@DOX10 effect on the morphology and size of TNBC tumor spheroids. As shown in Figure 9, a significant decrease in the size of tumor spheroids was apparent in the group treated with PAP@DOX10 compared to the control (PAP).

###  The effect of PAP@DOX on TNBC microtumor size 

**Figure 10:**
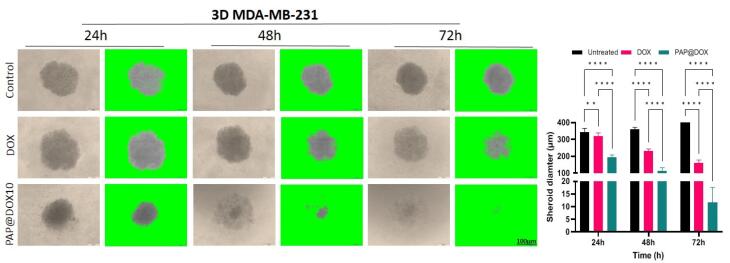
The effect of PAP@DOX on spheroid size and morphology

 We studied the anti-cancer potential of PAP@DOX on the size and morphology of large tumor spheroids for 24, 48 and 72 hours. In the control group, the spheroid size was increased from 344.78 ± 20.53 μm (24 hours) to 358.714 ± 13.13μm (48 hours) and reached the maximum size of 404.90 ± 14.90 μm within 72h. In the group treated with free DOX, the spheroid size was decreased to 320.3 ± 18.54 μm for 24 hours, and then 230.214 ± 13.06 μm for 48 hours and 159.64 ± 17.98 μm after 72 hours post-treatment. Finally, for the group treated with PAP@DOX10 the spheroid size was significantly dropped to 195.19 ± 12.61μm within 24 h, 111.64 ± 20.19 μm (48 hours) and 11.62 ± 5.97μm (72 hours) (Figure 10). Thus, PAP@DOX was capable of homogenous distribution and penetration into the core of hypoxic tumor spheroids.

###  The effect of PAP@TPZ on residual tumor cells 

**Figure 11:**
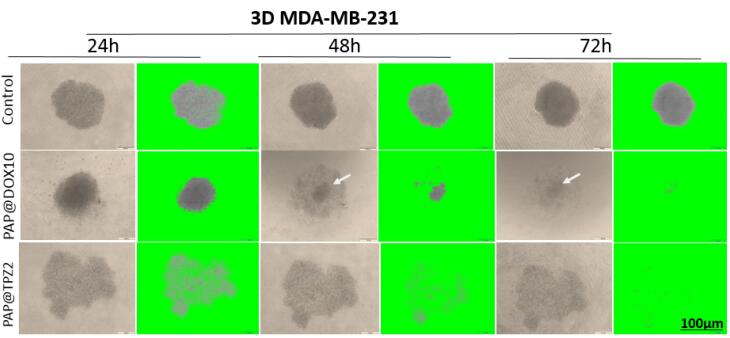
The effect of PAP@TPZ2 on residual tumor cells. Rim cells are shown with arrow

 Hypoxia created at the tumor site has been investigated and developed as a diagnostic and therapeutic target, due to the unique feature of solid tumors that is rarely observed in healthy tissue.^45^ Hypoxia-responsive prodrugs (HAPs) are now being examined in numerous clinical trials (see for review our recent paper^21^). RSU-1069, TH-302, TPZ and PR-104 are examples of clinically tested HAPs that specifically activate under hypoxic conditions and their optimum activity is enhanced by ~1000 times under hypoxic conditions. Furthermore, bioreductive agents like Nitroimidazoles (NIs) or their derivatives^46^ can be used as hypoxia-reducible crosslinkers for design of hypoxia-responsive NPs.^47^ With this in mind, in our study, we employed PAP@TPZ, which realized a two-step hypoxia-activatable NDDS, wherein the first stage, peg detachment occurs to expose highly-charged PAMAM NPs which swiftly penetrate the hypoxic tumor core. Then in the second phase, TPZ bioreduction into the TPZ radical occurs. Also, as treatment with PAP@DOX resulted in incomplete microtumor destruction, leaving a small fraction of viable cells in the tumor core (Figure 10), we sought to examine the therapeutic effect of a hypoxia-activated prodrug, TPZ. Surprisingly, unlike PAP@DOX10, PAP@TPZ2 treatment not only affected tumor edges, in line with enhanced activity of TPZ under hypoxia, but it also elicited the maximum potential on tumor rim cells and resulted in almost complete tumor eradication (Figure 11).

###  Quantitative RT real-time PCR

**Figure 12:**
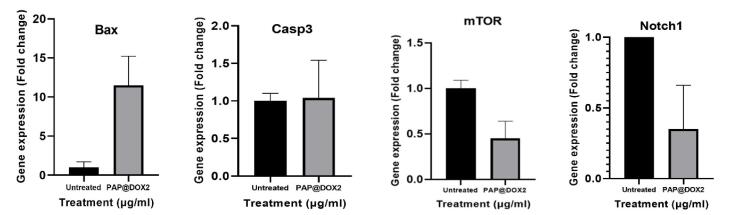
Gene expression analysis of key apoptosis and metastasis-related genes in TNBC model treated with PAP@DOX2

 To gain insight into the molecular mechanism of action of PAP@DOX, we analyzed the gene expression level of several key genes. We observed that treatment of TNBC tumor spheroids with PAP@DOX2 for 24 hours resulted in significant upregulation of BAX and Caspase3 by 11.51 and 1.049 folds compared to the control group. Also, for Notch1, and mTOR as key genes involved in the process of metastasis and stem cell formation, a significant decrease in mRNA levels was obtained at 2.78 and 2.21 folds compared to the control group, respectively (Figure 12).

 The efficacy of PAP@DOX was examined by 2D cell culture under normoxia (21% O_2_) and hypoxia (1% O_2_ level) conditions as well as 3D breast cancer mammospheres. As relevant biomimetics of tumor hypoxia,^48^ we find the superiority of 3D cell culture over 2D in terms of better replications of tumor hypoxia levels and thus monitoring the actual drug response. The enhancement in NP efficacy (cytotoxicity and ROS generation potential) in 2D cell culture under hypoxia compared to normoxia was barely 15%-20% as reported by Jang et al^49^ which reported only ~20% increment in DOX cytotoxicity under hypoxia *vs.* normoxia achieved by hypoxia-responsive folic acid conjugated glycol chitosan NPs upon A549 lung cancer cells. However, using large-size tumor spheroids, we find a sharp difference among the control and NP-treated groups regarding anti-cancer action. We were even able to monitor NPs distribution and its mechanism of action, which were capable of widespread and homogenous drilling starting from tumor outer layers and reaching tumor core by 72 hours. We also observed significant differences in gene expression in the level of key apoptotic genes of Bax, and caspase 3 genes as well as stemness and metastasis-promoting genes, Notch1 and mTOR. Surprisingly, we find interesting results using microtumor models’ systems regarding not only mechanism of anti-cancer therapeutic potential but also NP mode of distribution within hypoxic and well-perfused tumor regions. Such that irrespective of drug type, dendrimeric NP was highly efficient in widespread distribution and penetration into large mammospheres, however, we found that the effect of PAP@DOX treatment was clearly on tumor bulk focused on outer layers, meanwhile the hypoxic core was untouched even after 72 hours. This was surmounted by the use of TPZ which is specifically activated under hypoxia conditions and produced TPZ radicals. Thus, both vehicle (PAP) and drug (HAP) were synergized to achieve a dual-acting potential for targeting and eliminating both nearby and distant located tumor cells.

## Conclusion

 Together, herein we report on the design of a simple yet efficient nano drug formulation that possesses good biocompatibility, safety, and self-activating potential to deliver a variety of anti-cancer agents alone or in combination to the core of hard-to-reach solid tumors such as TNBC microenvironment.

## Acknowledgments

 This study is derived from MSc thesis of Mr. Mohammad Shahpouri and supported by Tabriz University of Medical Sciences (grant number: 65365).

## Competing Interests

 None to declare.

## Ethical Approval

 This study was approved by Tabriz University of Medical Sciences (ethical code: IR.TBZMED.VCR.REC.1399.456)
